# Evaluation of Knowledge among Dentists on Symptoms and Treatments of Temporomandibular Disorders in Italy

**DOI:** 10.3390/ijerph17238760

**Published:** 2020-11-25

**Authors:** Meysamian Mozhdeh, Francesco Caroccia, Francesco Moscagiuri, Felice Festa, Michele D’Attilio

**Affiliations:** Department of Medical, Oral and Biotechnological Sciences, University of Chieti-Pescara, 66100 Chieti, Italy; mozhdeh.meysamian@studenti.unich.it (M.M.); fcaroccia20@gmail.com (F.C.); francesco.moscagiuri@unich.it (F.M.); felice.festa@unich.it (F.F.)

**Keywords:** temporomandibular joint, temporomandibular disorders, knowledge, dentist

## Abstract

To determine the knowledge of general dentists and dental specialists on temporomandibular disorders (TMD) in Italy, a web-based questionnaire including 20 multiple- choice questions was sent to all general dental practitioners and specialists in Italy. Mean score of diagnosis and treatment knowledge of all participants was 23.8 ± 7.3 out of 40 achievable scores. There was a significant difference between the knowledge of dentists and the variables of sex, age, and years of experience (*p* < 0.05). However, overall, it is not possible to state a significant difference between the final score of dentists with different specializations (*p* = 0.89). The results of this study showed an acceptable knowledge of participants regarding TMD.

## 1. Introduction

The temporomandibular joint (TMJ), one of the most complex joints in the body, connects the mandible with the temporal bone of the skull [1]. The articular disk of this synovial joint allows sliding movements such that chewing, swallowing, and speaking can be effective and painless actions [2]. Temporomandibular disorders (TMDs) are frequent causes of cranio-facial diseases [3] which are characterized by pain, clicking, and discomfort during chewing and mouth opening [4]. It is worth noting that TMDs are very common disorders as almost 75% of the population has at least one sign of joint dysfunction (e.g., abnormal jaw movement, joint noises, tenderness on examination), and roughly 33% exhibits one or more symptoms of TMDs (joint and facial pain) [5,6]. Results of a study conducted in Italy have reported a high prevalence of TMDs in the adult population [7]. Pain associated with chronic forms of TMDs may negatively impact social and work activities of the individual, leading to an overall decrease in quality of life [8,9]. Dentists must know how to diagnose and treat TMDs, and if necessary, they should refer patients to TMD specialists. General dental practitioners (GDPs) are the first practitioner to be contacted and an effective and timely diagnosis can have important implications on the treatment of patients affected by TMD [10,11]. In order to evaluate the knowledge and convictions of GDPs with respect to TMDs, a number of studies have been carried out in different countries. Interestingly, a study in Spain (2019) indicated that general dentists appear to be good in defining disease etiology but not in its management [12]. An independent study performed in Turkey revealed that dentist knowledge on TMJ disorders and occlusal splint therapy is insufficient [13]. In 2010, an additional study conducted in Sweden reported that there was an urgent need to train orofacial pain/TMD experts who could, in turn, provide TMD continuing education to a large number of GDPs [14]. Finally, research in Teheran (Iran) confirmed the lack of an adequate TMD knowledge among dental practitioners [15]. Given the results of the above-mentioned studies showing that patients suffering TMD refer to general dental practitioners, and in consideration of the great prevalence of TMD, we analyzed the knowledge of dentists in Italy concerning TMD signs and treatment practices. The impact of several factors, including gender, age, degree, and years of practice, were also evaluated so that the outcomes could inform possible changes in education curricula, if needed.

## 2. Materials and Methods

In this cross-sectional study, a web-based questionnaire was sent by email to all dentists of the Abruzzo and Sicilia region listed in professional databases of ANDI (Associazione Nazionale Dentisti Italiani) and in groups composed by dentists on Facebook social media. A maximum of two reminders were sent by email within a period of 4 weeks. The study was conducted in observance of the Helsinki Declaration (revised version of Tokyo in 2004) and Good Clinical Practice Guidelines. The clinicians included in this study were informed about the purposes of the questionnaire and the anonymous nature of response. Dentists were subject to demographic questions, including age, gender, years of practice, and specialization, alongside 20 multiple-choice questions covering etiology, signs and symptoms, anatomy, diagnosis, and treatment, all selected from the Okeson reference textbook [1]. Each item was graded with 2 positive marks for each correct answer, and zero for each incorrect one. The final score was calculated by summing up the positive marks for each participant. To assess knowledge, the following classification was applied:

- scores 0–22: low
- scores 23–32: acceptable
- scores 33–40: high

SPSS software (version 24, SPSS, Chicago, IL, USA) was used to analyze the extracted data. The relationship between quantitative modules (e.g., experience and age) and subject knowledge was defined by using the Pearson’s correlation coefficient. For the gender, the Shapiro-Wilk test was applied while the Friedman test was used to evaluate the type of specialization. The level of statistical significance was expressed as a *p* value less than 0.05 (*p* < 0.05).

## 3. Results

Eighty-three dentists (46 men and 37 women) with an average age of 40 ± 12 years, and an average year of practice of 13 ± 11, filled in the questionnaires. From a 40 attainable score, the mean score of knowledge among dentists about diagnosis and treatment was 23.8 ± 7.3. The highest mean score of 25.3 ± 6.1 was associated with dentists who perform mainly conservative dentistry/endodontics treatments. Conversely, dentists with a specialty in Gnathology obtained the lowest average score of 20.9 ± 6.4. However, statistical analysis showed that it is not possible to claim an overall significant difference in the final score between dentists based on their specializations (*p* = 0.89). Most responsive dentists have worked in more than one specialty, so the test of several dependent communities (Friedman Test) has been used to test the effect of the type of specialty on the final score, as shown in Table 1.

The results appeared to be dramatically affected by several variables such as gender (*p* = 0.019), age (*p* < 0.001), and years of experience (*p* < 0.001). Interestingly, the knowledge of participants was shown to be inversely related with age and years of practice. A striking difference in knowledge was observed between men and women. The mean score of knowledge among women was 25.9 ± 6.7 and 22.2 ± 7.4 for the men. Twelve percent of subjects achieved a score higher than 33 points which means a high knowledge, 41%, resulted to have an acceptable knowledge (score between 23 and 32) and 47% resulted as low (score lower than 23). Questions and frequency of points earned from each question are reported in Table 2.

## 4. Discussion

TMD is highly prevalent and is known as the second most common cause of orofacial pain [16,17]. The vast majority of patients with TMD primarily refer to dentists. Thus, to correctly diagnose and effectively treat TMD, it is important that dentists acquire a comprehensive knowledge of the disease.

According to the findings reported in our study, 41% of the general dental practitioners had acceptable TMD knowledge, 12% were aware of TMD, and 47% showed an insufficient knowledge. Even though dentists whose specialty was conservative dentistry/endodontics (25.3 ± 6.1) showed higher knowledge and practitioners with gnathology (20.9 ± 6.4) specialty achieved a lower score in the questionnaire, no significant difference in TMD knowledge was observed between participants based on their specialty (*p* = 0.89). This questionnaire gave the possibility to choose more specializations as an answer. Results may therefore have been influenced by this factor. Since it is important to understand which professionals need more to improve their knowledge about TMD, it is necessary in future studies to formulate a questionnaire that will allow for selection of only the most practiced specialization. Indeed, since conservative dentistry is a common discipline in the daily practice of most professionals, it may also have been selected as an answer by specialists in other sectors.

By increasing age and years at practice, the knowledge dentists had about TMD was diminished. Female (25.9 ± 6.7) dentists showed better knowledge compared to male dentists (22.2 ± 7.4).

Many studies on the dentist knowledge of TMD were conducted in several countries.

In 2010, a study aimed at assessing the knowledge of general dental practitioners and specialists on TMD was performed in Tehran (Iran) [15]. According to the findings of this study, the knowledge was rated as following: low level (3%), relatively low level (72%), and fair (25%). It is worth noting that the Iranian study considered scores between 56% and 77% as “relatively low level,” while the current study defines similar scores as acceptable. Indeed, our study considered scores between 57.5% to 80% of correct answers as acceptable.

Results by Baharvand et al. [15] were in line with those of our study except for the interoperation, which was different. The questionnaire used by Baharvand et al. [15], included a section regarding dentists’ practice, which revealed that dentists with greater knowledge of TMD show greater tendency in managing such cases.

Moreover, Baharvand et al. [15] observed a decrease in the achieved score in relation to the growing age and practice experience of the participants. Similar results were also obtained by the current study [15].

A study performed in Seoul [18] also assessed the knowledge of dentists regarding TMD. The results of this study showed a good awareness of participants on psychiatric and psychophysiological disorders linked to TMD etiology. Yet, there was a considerable difference between participants and TMD experts regarding TMD pathophysiology knowledge, which included, among other things, diagnosis and management of chronic conditions [18]. Overall, results reported in this study were consistent with our findings in both diagnosis and treatment choices.

In 2015, an independent survey performed in Turkey [13] assessed knowledge, among dentists, concerning TMD and possible treatment approaches with occlusal splint. A total of 370 practitioners working in Turkey took part in the study. The results revealed that the knowledge of dentists was largely insufficient. Their knowledge diminished as years of professional practice increased [13]. This trend we also observed in our study as with increasing the experience, the participants’ knowledge was decreased. It is emerging that as the clinicians move away from the end of university studies, a progressive decline in theoretical knowledge happens. This result therefore suggests how important it is that professionals are offered periodical updating in general dentistry knowledge, and more specifically, in gnathology. Periodical updating can help clinicians remember fundamental notions firstly learned during their academic studies, and at the same time learn novel methodologies in the field. The goal of this continuous knowledge updating would be to offer patients high-quality therapeutic interventions.

TMD is highly prevalent in the population and represents a complex issue. Findings in the current study are open to interpretation due to the potentially rapid growth of knowledge on TMD in the days which were allotted for completion of the questionnaire. However, it may be stated that most dentists, particularly general dental practitioners, are not adequately prepared to diagnose and treat TMD.

Given that temporomandibular disorders are widespread among patients, it is of paramount importance that general dentists and even more orthodontists are adequately trained to be able to diagnose and solve these problems, as they are likely to be the first ones patients may consult.

Results presented in this study clearly show the urgency of strengthening professional curricula to better tackle complex medical issues related to diagnosis and treatment of a highly prevalent disease such as TMD. Comparisons between our study and those currently present in the literature also underline the fact that a gold standard questionnaire could be of great benefit for this type of research.

## 5. Conclusions

Based on the results of the current study, only half of participants had a proper knowledge (41% acceptable and 12% good) regarding TMD. The low percentage of clinicians who achieved a positive score in the questionnaire, appears to be inadequate for the effective management of patients affected by TMD in the country.

## Figures and Tables

**Table 1:** Final score according to clinician’s specialization.

	Mean	Std. Deviation	Minimum	Maximum
Conservative dentistry/Endodontics	25.3	6.1	16.0	36.0
Prosthetic dentistry	24.2	7.3	8.0	38.0
Periodontology	23.2	5.9	8.0	34.0
Oral surgery	23.3	6.6	2.0	34.0
Gnathology	20.9	6.4	2.0	34.0
Orthodontics	21.9	7.0	2.0	36.0

**Table 2:** Distribution of correct answers.

Question	Distribution of Correct Answers
How is maximum jaw opening measured, and where is the normal range of mouth opening located?	61.4%
How is “restricted mouth opening” defined?	84.3%
Which etiologic factors may be associated with the onset of temporomandibular disorder (TMD)?	92.8%
Is orthopedic instability the cause of TMD?	61.4%
Which type of premature contact is the most harmful one to the stomatognathic system?	62.7%
Where should the fingers be placed on the face to examine the temporomandibular joint (TMJ)?	48.2%
The condyle is normally positioned in … of the disk.	61.4%
The primary screening technique for TMD is …	66.3%
What is the standard gold survey for TMJ analysis?	59%
Can temporomandibular disorders lead to headache and orofacial pain?	45.8%
In which of the following conditions, the characteristic “click” at the temporomandibular joint is not produced?	54.2%
Which of these signs is associated with a joint block in the TMJ on the right side?	44.6%
Which of these signs is associated with a muscle block in the TMJ?	26.5%
What is the treatment choice when patients complain of a joint click in the absence of symptoms?	59%
Which of the following is a form of supportive therapy?	85.5%
Which medication may be used in TMD treatments?	84.3%
Suppose a patient with a previous history of joint click reports the spontaneous disappearance of the click, how should this condition be interpreted?	51.8%
If the right medial pterygoid muscle spasmodic …	45.8%
Are occlusal splints required in the treatment of TMD?	48.2%
Is physical therapy useful in the treatment of TMD?	50.6%
